# Implementation of adaptive integration method for free energy calculations in molecular systems

**DOI:** 10.7717/peerj-cs.264

**Published:** 2020-03-16

**Authors:** Christopher A. Mirabzadeh, F. Marty Ytreberg

**Affiliations:** 1 Department of Physics, University of Idaho, Moscow, ID, United States of America; 2 Institute for Modeling Collaboration and Innovation, University of Idaho, Moscow, ID, United States of America; 3 Institute for Bioinformatics and Evolutionary Studies, University of Idaho, Moscow, ID, United States of America

**Keywords:** Adaptive integration, Monte Carlo, Free energy, Solvation, Protein, Biomolecule

## Abstract

Estimating free energy differences by computer simulation is useful for a wide variety of applications such as virtual screening for drug design and for understanding how amino acid mutations modify protein interactions. However, calculating free energy differences remains challenging and often requires extensive trial and error and very long simulation times in order to achieve converged results. Here, we present an implementation of the adaptive integration method (AIM). We tested our implementation on two molecular systems and compared results from AIM to those from a suite of other methods. The model systems tested here include calculating the solvation free energy of methane, and the free energy of mutating the peptide GAG to GVG. We show that AIM is more efficient than other tested methods for these systems, that is, AIM results converge to a higher level of accuracy and precision for a given simulation time.

## Introduction

Measuring free energy differences using computer simulations can be computationally expensive, yet is useful for many different applications (see e.g., Steinbrecher & Labahn, 2010; Chodera et al., 2011; Mobley et al., 2012; Zhan et al., 2013; Miller et al., 2014; Petukh, Li & Alexov, 2015; Zhan & Ytreberg, 2015; Miller et al., 2016; Cournia, Allen & Sherman, 2017; Hossain et al., 2019; Aminpour, Montemagno & Tuszynski, 2019). Specific examples include determining protein conformational preferences, virtual screening for drug design or drug discovery (Steinbrecher & Labahn, 2010; Chodera et al., 2011; Zhan & Ytreberg, 2015; Śledź & Caflisch, 2018; Aminpour, Montemagno & Tuszynski, 2019; Zhang et al., 2019). Of specific relevance to the current study is that free energy calculations allow prediction of how amino acid mutations may modify protein-protein binding (Zhan et al., 2013; Miller et al., 2014; Petukh, Li & Alexov, 2015; Miller et al., 2016; Geng et al., 2019). We are particularly interested in developing and implementing efficient methods for calculating free energy differences and using them to understand how amino acid mutations modify protein–protein and protein-substrate interactions.

For this study, we have implemented the adaptive integration method (AIM) introduced by Fasnacht, Swendsen & Rosenberg (2004) for use in the GROMACS (Berendsen, Van der Spoel & Van Drunen, 1995) molecular dynamics simulation package, and have compared results to a suite of other methods. In previous studies, AIM was shown to provide high quality, precise and efficient estimates of binding free energies (Ytreberg, Swendsen & Zuckerman, 2006; Kaus, Arrar & McCammon, 2014; Kaus & Mccammon, 2015). We focus on alchemical free energy calculation where a system is transformed from one state to another via an unphysical pathway. The progress along the pathway that connects the two states is defined by the parameter *λ*. For this study, we are interested in two ways to explore *λ* space—both of which have the goal of obtaining equilibrium sampling of system configurations at discrete *λ* values along the pathway. The first way is to treat *λ* as a system variable that can be biased and sampled. A class of such methods, termed generalized ensemble, use an extended Hamiltonian to sample *λ* (Bitetti-Putzer, Yang & Karplus, 2003). For example, *λ*-dynamics (Kong & Brooks, 1996; Knight & Brooks, 2009; Knight & Brooks, 2011) treats *λ* as a dynamic particle in the system with fictitious mass. By contrast, AIM uses Metropolis Monte Carlo to sample *λ* space (Fasnacht, Swendsen & Rosenberg, 2004). Monte Carlo moves between values of *λ* are based on running estimates of free energy differences; this is a key distinction from other methods and allows AIM to continuously improve the estimate for the free energy during the simulation. The second way to explore *λ* space is to perform standard molecular dynamics or Monte Carlo simulations at fixed values of *λ*, typically discarding some simulation time for equilibration. The configurational ensembles at each value of *λ* can then be used to estimate free energy differences (see e.g., Lyubartsev, Førrisdahl & Laaksonen, 1996; Gonçalves & Stassen, 2004; Kofke, 2005; Shirts, Mobley & Chodera, 2007; Chodera & Shirts, 2011; Klimovich, Shirts & Mobley, 2015). In order to provide comparisons for AIM to fixed *λ* methods, we used the Python tool alchemical-analysis.py (Klimovich, Shirts & Mobley, 2015), part of the Pymbar package (Shirts & Chodera, 2008). This tool estimates the free energy using a suite of methods such as the Bennett acceptance ratio, multistate Bennett acceptance ratio, thermodynamic integration and exponential averaging.

For the current study, we chose two molecular systems that have well-documented results and are important starting points for biomolecular free energy studies. First, we calculated the solvation free energy of methane. Simulations were performed and the free energies were calculated using the fixed *λ* methods provided by alchemical-analysis. Simulations were also performed using AIM and results compared to fixed *λ* simulations. Using the lessons learned from the methane system, we then calculated the free energy of mutating the peptide GAG to GVG in water. For both systems, we found that AIM produces free energy estimates that are within statistical uncertainty of fixed *λ* methods but with greater efficiency (i.e., more accurate for a given simulation time).

## Methods

All methods, code and simulation input files are available in the supplemental materials. For this study, we performed alchemical free energy simulations where the system is changed from a reference state to an end state by constructing a reaction pathway that modifies, adds or removes atoms. Such alchemical simulations are non-physical, i.e., the simulation does not represent what could occur naturally. Since the free energy is a state variable, it is independent of the path taken, and we may provide any path we wish. To perform these simulations the reaction pathway is divided into many separate, non-physical, *λ* states between a reference state and an end state. The *λ* states represent the progress along the reaction pathway as the reference state transforms into the end state.

Like most methods used to calculate free energies we start from the identity,

(1) \begin{eqnarray*}F=U-TS,\end{eqnarray*}

where *U* is the potential energy, *T* is the temperature and *S* is the entropy of the system. For free energy differences we generalize the formulation of the change in free energy by separating calculations into two, non-overlapping, thermodynamic end states, *A* and *B*, at constant system temperature *T*,

(2) \begin{eqnarray*}\Delta F\equiv \Delta {F}_{A\rightarrow B}={F}_{B}-{F}_{A}=\Delta U-T\Delta S.\end{eqnarray*}

Δ*F* is the change in free energy, Δ*U* is the change in potential energy and Δ*S* is the change in entropy of the system. According to statistical mechanics, the free energy difference between the two end states, *A* and *B*, of the system is the log of the ratio of the configurational partition functions (see discussion in Chipot & Pohorille (2007)),

(3) \begin{eqnarray*}\Delta F=-{k}_{B}T\ln \nolimits \frac{Z[{U}_{B}(\vec{x})]}{Z[{U}_{A}(\vec{x})]} .\end{eqnarray*}

Here, *k*_*B*_ is the Boltzmann constant and $Z[U(\vec{x})]$ is the configurational partition function for the energy states ${U}_{A}(\vec{x})$ and ${U}_{B}(\vec{x})$, where $\vec{x}$ is the vector of configuration coordinates. The configurational partition function is given by

(4) \begin{eqnarray*}Z[U(\vec{x})]=\int \nolimits \exp \nolimits (-\beta U(\vec{x}))d\vec{x},\end{eqnarray*}

and $\beta = \frac{1}{{k}_{B}T} $.

Computationally, we calculate free energy differences between end states by performing molecular dynamics simulations along a reaction pathway of intermediate states, defined by *λ*, such that, 0 ≤ *λ* ≤ 1.

This pathway connects the two end states of the system. In the case of poor overlap, where the end states may be separated by a high energy barrier, |*U*_*B*_ − *U*_*A*_| ≫ *k*_*B*_*T*, this pathway mitigates the otherwise very slow convergence of free energy estimates (Shirts, Mobley & Chodera, 2007). Care should be taken when choosing intermediate states such that there is adequate overlap in the conformation space between the end states (Shirts, Mobley & Chodera, 2007; Klimovich, Shirts & Mobley, 2015). For our simulations the number of *λ* values and time per *λ* were chosen through extensive trial and error (more on this below).

The method of exponential averaging (DEXP, IEXP) (Zwanzig, 1954) starts from Eq. (3) above and then adding and subtracting $\exp (-\beta U(\vec{x}))$ from the integral in the configurational partition function of the numerator we end up with the final relationship,

(5) \begin{eqnarray*}\Delta {F}_{ij}=-{k}_{B}T\ln \nolimits \langle \exp \nolimits (-\beta \Delta {U}_{ij}(\vec{x}))\rangle _{{\lambda }_{i}}.\end{eqnarray*}

where Δ*F*_*ij*_ is the free energy between *λ*_*i*_ and *λ*_*j*_ and 〈⋅〉_*λ*_*i*__ represents an average of the equilibrium configuration for *λ*_*i*_. Unlike some other methods, exponential averaging has an exact solution since it is only used to evaluate the difference between two states. However, it is the least efficient method and should not be used if difference in potential energies are much larger than *k*_*B*_*T* (Shirts & Pande, 2005). In addition, exponential averaging can be noisy, biased and dependent on the tails of the distribution of *λ* states (Bruckner & Boresch, 2011; Shirts & Pande, 2005).

For thermodynamic integration (TI) we estimate the free energy by first looking at the derivative of Eq. (1) with respect to *λ*,

(6) \begin{eqnarray*} \frac{\partial F}{\partial \lambda } ={ \left\langle \frac{\partial U}{\partial \lambda } \right\rangle }_{\lambda }.\end{eqnarray*}

This differential equation, Eq. (6), can then be integrated to give,

(7) \begin{eqnarray*}\Delta F=\int \nolimits \nolimits _{\lambda =0}^{1}{ \left\langle \frac{\partial {U}_{\lambda }(\vec{x})}{\partial \lambda } \right\rangle }_{\lambda }d\lambda \end{eqnarray*}

where the 〈⋅〉_*λ*_ notation represents the ensemble average at a given intermediate state, *λ*. The free energy is estimated by numerically integrating Eq. (7) after running equilibrium simulations at each intermediate *λ* state. Since numerical integration is required, TI can be biased by the chosen method of integration. Some of that bias can be removed by using cubic-spline interpolation or more complex integration estimators(Shirts & Pande, 2005; Shyu & Ytreberg, 2009).

The Bennett (Bennett, 1976) and multistate (Shirts & Chodera, 2008) Bennett acceptance ratio (BAR and MBAR) methods are far more efficient than exponential averaging and are commonly used to avoid the shortcomings of other methods (Shirts & Pande, 2005; Ytreberg, Swendsen & Zuckerman, 2006). BAR and MBAR typically achieve the same statistical precision as TI with fewer *λ* states unless the integrand for TI is very smooth (Shirts & Mobley, 2013; Ytreberg, Swendsen & Zuckerman, 2006). The complete derivation can be found in Bennett’s paper (Bennett, 1976) but the premise is; for sufficiently large samples *n*_*i*_ of *U*_*i*_ and *n*_*j*_ of *U*_*j*_,

(8) \begin{eqnarray*}\Delta F(i\rightarrow j)={k}_{B}T\ln \nolimits \frac{\langle f(\Delta {U}_{ij}+C)\rangle _{j}}{\langle f(\Delta {U}_{ji}-C)\rangle _{i}} +C.\end{eqnarray*}

C is a shift constant,

(9) \begin{eqnarray*}C={k}_{B}T\ln \nolimits \frac{{n}_{j}}{{n}_{i}} ,\end{eqnarray*}

and *f*(*x*) is the Fermi function,

(10) \begin{eqnarray*}f(x)= \frac{1}{1+\exp \nolimits (\beta x)} .\end{eqnarray*}

Equation (8) is the ratio of canonical averages of two different potentials *U*_*i*_ and *U*_*j*_ acting on the same configuration space meaning it requires information from two neighboring states. However, this limitation is not too much of a concern with a trivial coordinate transformation or when using dummy coordinates in alchemical simulations. MBAR, an extension of BAR, differs in that it takes data from more than two states hence the name “multistate”.

AIM is similar to TI in that numerical integration of Eq. (7) is performed; the key difference is how the averages ${ \left\langle \partial U/\partial \lambda \right\rangle }_{\lambda }$ are obtained. AIM uses Metropolis Monte Carlo to move in *λ* space and ordinary running averages are calculated at each *λ* value. In AIM, a random move from *λ*_*old*_ to *λ*_*new*_ is accepted with probability

(11) \begin{eqnarray*}\min \nolimits \{1,\exp \nolimits (-\beta ({U}_{new})-{U}_{old})+\beta ({F}_{new}-{F}_{old})\}\end{eqnarray*}

where *U*_*new*_ − *U*_*old*_ is the difference in the potential energy for the old and new *λ* values. *F*_*new*_ − *F*_*old*_ is the estimated free energy difference based on the current running averages of ∂*U*∕∂*λ*.

### Implementation

AIM was implemented in GROMACS as an expanded ensemble calculation. That is, the Hamiltonian must be calculated along with its derivative, and an expanded ensemble step must be performed for every dynamics step. In GROMACS, nstexpanded is the number of integration steps between attempted *λ* moves changing the system Hamiltonian in expanded ensemble simulations. This value must be a multiple of nstcalcenergy, the number of steps before calculating the system energy, but can be greater or less than nstdhdl, the number of steps before calculating ∂*U*∕∂*λ* (referred to as dHd*λ* in GROMACS documentation). For a detailed explanation of all technical terms see reference Abraham et al. (2016). The GROMACS package was further altered to print out the ∂*U*∕∂*λ* averages computed by AIM to the log file when AIM is used as the lmc-mover.

AIM requires the ∂*U*∕∂*λ* value from every dynamics step to be stored regardless of whether a move in *λ* space is attempted. Since ∂*U*∕∂*λ* is only calculated at each step where free energies are calculated, every nstdhdl step, we set nstexpanded = nstdhdl = nstcalcenergy = 1 for AIM simulations. This further implies that lmc-stats functions were not used during AIM simulations because those functions modify the Hamiltonian which is not needed for AIM.

For the implementation of AIM with GROMACS we follow the outline given in our previous study Ytreberg, Swendsen & Zuckerman (2006).

1. Start the simulation from an equilibrated configuration at *λ*=0 and perform one molecular dynamics step.
2. Randomly choose a trial move in *λ* space. For example, if our *λ* spacing is 0.05, a move from *λ* = 0.35 to 0.4 or 0.3 may be attempted but not to 0.45.
3. Calculate the difference in potential energy between the trial and current *λ* values.
4. Estimate the free energy difference between the trial and current *λ* values using the running averages of ∂*U*∕∂*λ* and the trapezoidal rule.
5. Accept *λ* trial with probability given in Eq. (11).
6. If the move is accepted then *λ* is updated to the trial value, otherwise the simulations stays at the current *λ*.
7. The running average of ∂*U*∕∂*λ* is updated.

### Simulation details

The first system used here, methane in water, is detailed in systematic studies of force fields and the free energies of hydration of amino acid side chain analogs (Sun et al., 1992; Lyubartsev, Førrisdahl & Laaksonen, 1996; Chodera & Shirts, 2011; Paliwal & Shirts, 2011). For the GAG to GVG mutation the PMX (Gapsys et al., 2015) software package was used to construct the tri-peptide mutation. Using PMX, we generated the hybrid protein structure and topology for simulations of the chosen mutation, alanine to valine.

All simulations described in this paper were performed using the molecular dynamics package GROMACS 5.1.4. The simulations were carried out at 300 K and solvated in a dodecahedron box with TIP3P waters. The methane molecule was parameterized using the OPLS (Optimized Potential for Liquid Simulations) force field (Jorgensen, Maxwell & Tirado-Rives, 1996). The OPLS force field was chosen for this study because it is known to perform well on small molecules (Shirts et al., 2003). In future studies, we anticipate using AIM on protein systems where other force fields are more appropriate such as AMBER (Salomon-Ferrer, Case & Walker, 2013) and CHARMM (Mackerell, Banavali & Foloppe, 2001). Since all molecular dynamics force fields have similar form and number of parameters, it is expected that the performance of AIM would not depend on the force field chosen. For the GAG to GVG mutations, Na+ and Cl- ions were added to keep the simulation box neutral and reach a physiologically relevant 150 mM salt concentration.

For both systems, energy minimization was performed using steepest descent for 1,000 steps. The system was then equilibrated using simulated annealing for 1,000 ps to heat the system from 100 K to 300 K. For production simulations, electrostatic interactions were handled by Reaction field with a cut-off of 0.9 nm, Potential-shift-Verlet modifier and Verlet cutoff scheme. Van der Waals interactions were handled by twin range cutoffs with neighbor list cutoff of 1.15 nm and van der Waals cutoff of 0.9 nm. The bonds involving hydrogens were constrained with the Shake algorithm, allowing for a 2 fs time step. Long range dispersion corrections for energy and pressure were applied. For the free energy calculations, softcore scaling was used with parameters sc-power =1, sc-r-power =6 and sc-alpha =0.5. In addition, the van der Waals and Coulomb interactions were separately turned on or off as a function of *λ*. That is, one is held fixed as a function of *λ* while the other changes. For the methods processed with alchemical-analysis.py we ran fixed *λ* simulations. That is, an equal amount of simulation time was spent at each *λ* value. For AIM we ran expanded ensemble simulations where we alternate between taking molecular dynamics steps and attempting trial moves in *λ* space. That is, for AIM the amount of time spent at each *λ* value is determined by the algorithm.

In order to determine the best distribution of intermediate *λ* states we followed a simple strategy: (i) Conduct short simulations with a small set of intermediates. (ii) Generate a plot comparing slope values between AIM and fixed *λ* (iii) Determine the locations of curvature in the estimate of the free energy. (iv) Increase the density of intermediate states in locations of high curvature. (v) Repeat until all areas of high curvature have been well explored. We note that these steps should be performed for any method where the slope of the free energy is used to calculate free energies, including both TI and AIM. Similar steps would be performed for methods such as DEXP, IEXP, BAR and MBAR to ensure that energy differences are not too large between *λ* values.

## Results

### Methane

**Figure 1:**
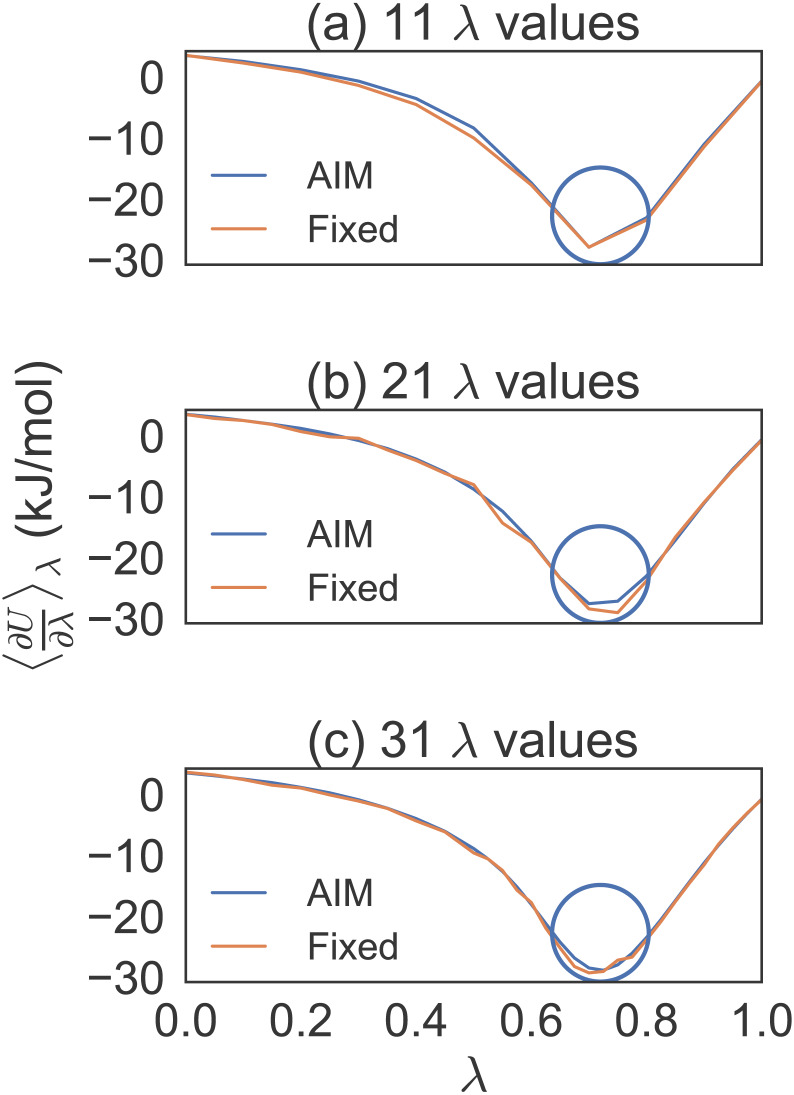
Different *λ* densities for methane solvation free energy calculations. Eight trial simulations of 100 ps per *λ* for 11, 21 and 31 *λ* values. This shows how the number of *λ* values were chosen to effectively compare AIM to fixed *λ* simulations. The circles indicate the region where the *λ* density needed to be increased.

After conducting short simulations, generating plots to determine locations of high curvature and increasing *λ* density in those regions, we averaged eight trial simulations of 100 ps per *λ* for separate *λ* distributions (see Fig. 1). We found, by progressively increasing the *λ* density between *λ* = 0.5 and *λ* = 1.0, that a distribution of 31 *λ* values gave us a dense enough distribution to properly compare AIM to fixed *λ* methods for the methane simulations.

**Figure 2:**
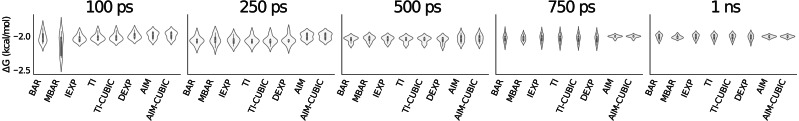
Violin plot showing methane solvation results for 31 *λ* values averaged over eight trials. A violin plot combines a box plot and a density plot to visualize the distribution and probability density. The graphic shows all methods have similarly converged at 1 ns per *λ*. AIM and AIM-CUBIC converge earlier than other methods at 750 ps per *λ*.

Figure 2 is a violin plot to visualize the distribution and probability densities over the eight trials for each method as a function of simulation time per value of *λ*. A violin plot combines a box plot and a density plot to show the shape of the distribution around the mean. The thick black bar in the center represents the interquartile range, the white dot is the median and the thin black line going vertically through the middle represents the upper and lower adjacent values. Reading a violin plot is similar to reading a density plot. The thicker parts represent high frequency values and the thinner parts represent low frequency values. The advantage of a violin plot over a box plot is that we are able to view the underlying distribution of the data.

In Fig. 2 at 100 ps per *λ* most methods have similar standard deviations of around 0.05 kcal/mol (visualized by the height of the violin shape in the figure), but the slower convergence of MBAR in this case leads to a larger standard deviation of around 0.16 kcal/mol. By 750 ps AIM has converged to a smaller standard deviation of around 0.02 kcal/mol compared to the other methods at around 0.05 kcal/mol.

### GAG to GVG Mutation

**Figure 3:**
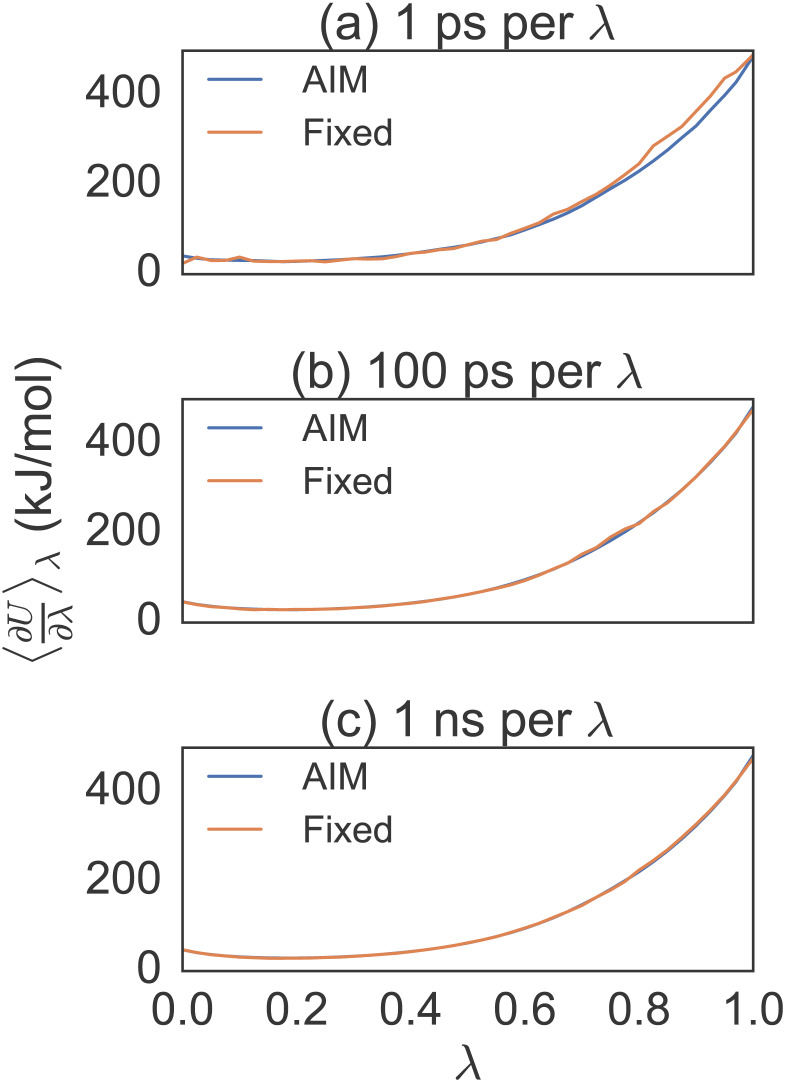
Different simulation times for alanine to valine mutation free energy calculations. Eight trial simulations of 41 *λ* values at 1 ps, 100 ps and 1 ns per *λ*. Note the smoothness of AIM versus fixed *λ* simulations. AIM requires less samples than fixed *λ* simulations to smooth the free energy function.

**Figure 4:**
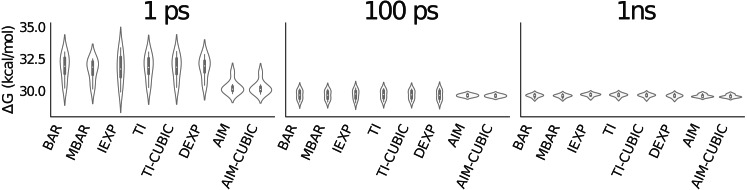
Violin plot showing alanine to valine mutation results for 41 *λ* values averaged over eight trials. The graphic shows all methods have similarly converged at 1 ns per *λ*. AIM and AIM-CUBIC converge more rapidly than other methods and are mostly converged at 100 ps per *λ*.

For the GAG to GVG mutation we first tested a distribution of 41 *λ* values averaged over 8 trial simulations of 1 ps and 100 ps per *λ*; see Fig. 3. By reviewing the smoothness of the function we concluded that 41 *λ* values was sufficient. Figure 4 shows the convergence of the free energy for GAG to GVG over time for each method. At just 1 ps per *λ* value the AIM estimates are within less than 1.0 kcal/mol of the converged (1 ns per *λ*) result with a standard deviation of around 0.5 kcal/mol. All other methods are more than 2.0 kcal/mol from this converged result with larger standard deviations of around 1.0 kcal/mol. At 100 ps all estimates are less than 0.1 kcal/mol from the converged result, but AIM estimates have a standard deviation of around 0.1 kcal/mol compared to other methods at 0.4 kcal/mol. All methods have similarly converged at 1 ns per *λ*.

## Discussion

In the limit of infinite sampling, all rigorous methods (i.e., statistical mechanics-based methods), performed properly with the same force-field and parameters, will yield the same result within uncertainty. Often, it is of interest to define accuracy by comparing results to experimental data. However, given that the purpose of this study is to compare various computational methods, we define accuracy by comparing results to the value upon which all methods converge. For fixed *λ* simulations the sampling time is typically the same for each *λ* state. Sampling time must be increased whenever convergence has not been achieved. However, if bias is introduced by using an insufficient number of *λ* values in regions of high curvature, increased sampling leads to radical convergence problems (Shyu & Ytreberg, 2009; Steinbrecher & Labahn, 2010). If the curvature of the underlying free energy slope values is large, averaging over a state space that is not dense enough to fully describe the state function propagates this bias requiring significantly increased sampling time to achieve convergence. For TI, the bias will persist even for infinite sampling. In addition, increasing sampling time may not be realistic when dealing with limited computational resources. Paliwal & Shirts (2011) make a detailed argument to why convergence may not be possible for all systems due to hard limitations in computational resources.

In particular, both TI and AIM are calculating the same slope averages and should agree very well for simple systems and reasonably long simulation times. However, before convergence is reached, due to the fact that AIM spends more time in some regions, we should not expect the approximation of AIM to exactly match TI with similar sampling time until the number of *λ* values has been sufficiently increased in high curvature regions. Once we have properly chosen the *λ* values then reasonably long simulations will lead to highly similar results between these two methods.

AIM is able to estimate the free energy for the amino acid mutation within 1.0 kcal/mol at a total simulation time of only 41 ps. This is quite remarkable since ±1.0 kcal/mol is typically the range that is desired for mutation studies. Of course, further studies using a broader range of amino acid change are needed, but it suggests that AIM may be suitable for quick estimation. We believe the reason that AIM performs so well in such cases is due to the Monte Carlo sampling that allows AIM to more efficiently sample *λ* space compared to fixed *λ* simulations.

The reader may note that AIM violates detailed balance since the acceptance criterion contains the free energy estimates that are updated continuously. AIM does however obey detailed balance asymptotically. As simulation time increases, the average free energy differences between *λ* values reach an equilibrium and detailed balance is satisfied. Once this equilibrium is attained the algorithm will sample all *λ* values equally, that is, the histogram of the number of configurations will become flat as a function of *λ*.

## Conclusion

In this report we have implemented the adaptive integration method (AIM) for calculating free energy differences in GROMACS and applied it to two molecular systems. We have shown agreement within statistical uncertainty between AIM and a suite of fixed *λ* methods for methane solvation and an GAG to GVG mutation. We have also shown that AIM is more efficient than the other tested methods. That is, for a given amount of simulation time, AIM has a higher level of accuracy and precision. We anticipate these findings will extend to larger, more complex systems. Future studies will be performed to test whether this is the case.

Further, we found that running longer simulations with too few intermediate *λ* states generated results that were inconsistent between methods. The density and sampling convergence of the *λ* states directly influences the agreement between all the tested methods. Since some states will contribute disproportionately to the variance of the estimate, we found that generating short test simulations of different *λ* densities before attempting longer simulations is advisable.

##  Supplemental Information

10.7717/peerj-cs.264/supp-1 Supplemental Information 1 Source code, input file, and Jupyter notebook Three directories: (1) gmxProgFiles contains source code to build AIM into GROMACS 5.1.4; (2) gmxInputFiles contains input files for GROMACS to reproduce our results; (3) AnalysisFiles contains Jupyter notebook files for analyzing results producing graphs.

## Additional Information and Declarations

### Data Availability

The following information was supplied regarding data availability: All code used to generate the results, including scripts and modified GROMACS code are available in the Supplemental Files.
